# The influence of 5-HTTLPR and Val66Met polymorphisms on cortical thickness and volume in limbic and paralimbic regions in depression: a preliminary study

**DOI:** 10.1186/s12888-016-0777-x

**Published:** 2016-03-15

**Authors:** Natalia Jaworska, Frank P. MacMaster, Jane Foster, Rajamannar Ramasubbu

**Affiliations:** Department of Psychiatry, McGill University, Montreal, PQ Canada; Department of Psychiatry, Mathison Centre for Mental Health Research & Education, University of Calgary, #4D64 TRW Building, 3280 Hospital Drive NW, Calgary, AB T2N4Z6 Canada; Hotchkiss Brain Institute, University of Calgary, Calgary, AB Canada; Child & Adolescent Imaging Research (CAIR) Program, Alberta Children’s Hospital Research Institute for Child & Maternal Health, Calgary, AB Canada; Department of Psychiatry & Behavioral Neurosciences, McMaster University, Hamilton, ON Canada

**Keywords:** Cortical thickness, Structural volume, Depression, Val66Met, 5-HTTLPR, Polymorphism

## Abstract

**Background:**

Structural brain abnormalities have been investigated in multi-genetic and complex disorders such as major depressive disorder (MDD). Among the various candidate genes implicated in MDD, the brain-derived neurotrophic factor (BDNF) Val66Met polymorphism and 5-HT transporter gene linked polymorphism (5-HTTLPR) have garnered the most attention due to their putative roles in neural plasticity and antidepressant response. However, relatively few studies have assessed the influence of these polymorphysims on cortical thickness or brain volume in para-limbic and limbic regions in MDD, which was the aim of this study.

**Methods:**

Forty-three adults with MDD and 15 healthy controls (HC) underwent structural magnetic resonance imaging (MRI). Cortical thickness was assessed in frontal, cingulate and temporal regions. Volumetric measures were carried out in the thalamus, caudate, putamen, pallidum, hippocampus and amygdala. Participants were genotyped to determine their 5-HTTLPR (tri-allelic) and Val66Met polymorphisms.

**Results:**

In the combined sample (MDD + HC), smaller right pallidum volumes were found in L_A_/S (L_A_/S & L_A_/L_G_) heterozygotes compared to S/S (S/S, L_G_/S & L_G_/L_G_) homozygotes, though the effect was modest. In the MDD group, larger left thalamus and putamen volumes were observed in L_A_/L_A_ homozygotes. No Val66Met or 5-HTTLPR genotype effects existed on cortical thickness and no main effects of the Val66Met polymorphism were observed.

**Conclusion:**

Our preliminary results suggest that the 5-HTTLPR polymorphism is associated with morphometric changes in regions known to play an important role in emotional and reward processing in depression. A larger sample size is required to replicate these findings and to potentially reveal subtle morphometric changes.

**Electronic supplementary material:**

The online version of this article (doi:10.1186/s12888-016-0777-x) contains supplementary material, which is available to authorized users.

## Background

The etiology of major depressive disorder (MDD) is complex - encompassing social, environmental, physiological and genetic factors. While accumulating data indicate that no single gene is substantially implicated in increasing MDD risk [1], two genes that have been extensively studied and linked with the disorder include those related to the expression of brain-derived neurotrophic factor (BDNF) and the serotonin transporter (5-HTT). Intermediate phenotypes, such as abnormalities in brain structure/morphometry, can be conceptualized as manifestations linking genetic factors and overt disorder indices. The majority of research has focused on examining volumetric changes in para/limbic structures, such as the hippocampus [2], in MDD; cortical thickness assessments in MDD are less common. However, cortical thickness, which reflects neuronal size, density and arrangement, as well as those of neuroglia and nerve fibers, can provide unique information about disorder-specific neuroanatomical features [3]. Given that regional cortical thickness has been linked with specific cognitive domains [4], and allowing for the possibility that subtle cortical thickness changes may be easier to capture than gross structural volume measures, a more thorough assessment of this metric in the context of mental illness is warranted.

Accumulating evidence suggests that depressed adults (including elderly depressed individuals) tend to exhibit diffuse cortical thinning, including in frontal regions [medial orbitofrontal (OBF) cortex, dorsolateral prefrontal cortex (DLPFC)], insula, cuneus, posterior cingulate and middle temporal cortices [5–12], though exceptions exist [13, 14]. On the other hand, although we previously noted thinner transverse temporal cortices, we also found thicker frontal pole cortices in depressed adults [15]. Another group also reported thicker cortices in the right frontal lobe (medial OBF, pars opercularis, rostral middle frontal gyrus) and supramarginal gyrus in treatment-naïve adults with first-episode MDD [3]. A handful of studies in pediatric/adolescent MDD have reported both cortical thinning (e.g. pericalcarine, post-central, superior parietal/supramarginal gyrus regions) and thickening (e.g. temporal poles, middle frontal gyrus, caudal cingulate gyrus) [16, 17]. Work by our group and others suggest that factors such as age of MDD onset and trauma history may effect cortical thickness [5, 15]. Genetic influences on cortical thickness in MDD, however, have scarcely been examined.

### Val66Met polymorphysim: influence on brain architecture

Most research has focused on the influence of the valine (Val)-to-methionine (Met) substitution at codon 66 (Val66Met) in the BDNF gene; this polymorphysim is believed to affect BDNF intracellular trafficking and decrease activity-dependent secretion [18]. Met allele carriers appear to be at greater risk for MDD development when exposed to stressful life events [19]. Further, converging data suggest decreases in hippocampal volumes in Met (vs. Val/Val) allele carriers in both controls and MDD patients [20–25] (but see [26, 27]), though one group found that this was driven by the MDD cohort [28] while another reported that this was mediated by childhood adversity [29]. Volumetric decreases in relatively diffuse brain regions have also been reported in non-depressed Met (vs. Val/Val) carriers [30–32]. However, the role of the Val66Met polymorphisms on non-hippocampal volumetric brain changes and cortical thickness in the context of MDD remains unclear.

### 5-HTTLPR polymorphysim: influence on brain architecture

In comparison to the Val66Met polymorphism, research on the role of the base-pair repeat polymorphism in the promoter region of the 5-HTT (5-HTTLPR) in MDD is more extensive. The 5-HTTLPR polymorphism lies in the promotor region of the 5-HTT encoding gene (SLC6A4), with a 44 base-pair (bp) insertion/deletion generating two 5-HTTLPR alleles: S (14 bp repeats) and L (16 bp repeats) [33]. Further, an A/G nucleotide substitution in the L allele (rs25531) renders the 5-HTTLPR tri-allelic (L_G_/L_G_, L_G_/L_A_, L_A_/L_A_); the L_G_ is purported to be functionally similar to the S allele [34]. Studies suggest that the L (vs. S) allele may have higher transcriptional activity, which affects synaptic 5-HT clearance [35]. A complex relation appears to exist between the 5-HTTLPR polymorphism and MDD risk, with S (vs. L) allele carriers having a greater risk for MDD development in the context of adversity [36]. Surprisingly little research has examined the effects of the 5-HTTLPR polymorphism (tri- or diallelic) on cortical thickness/brain morphometry.

Existing data on 5-HTTLPR polymorphism effects on brain morphometry are mixed. Frodl et al., found grey matter decreases in L_A_/L_A_ MDD patients in the amydgala, hippocampus, anterior cingulate cortex (ACC) and aspects of the dorsomedial PFC and DLPFC; in controls, the opposite was found [37]. On the other hand, when using a diallelic classification, another group reported smaller hippocampi in S/S MDD patients (vs. controls); no differences existed between controls and patients who were S/L heterozygotes or L/L homozygotes [38]. However, this relation may be sex-dependent as others noted that non-depressed S allele carrying females had larger hippocampi than L/L females while the opposite was found in males [39]. With respect to other brain regions, Selvaraj et al., reported reduced gray matter volumes in the inferior frontal gyrus, ACC and superior temporal gyrus in non-depressed S allele carriers relative to L/L homozygotes [40]. Yet another, however, found that S allele carriers versus L/L homozygotes had greater amygdala volumes irrespective of diagnosis (MDD or control) [41]. Finally, a handful of studies have assessed the putative interactive (epistasis) effects between the Val66Met and 5-HTTLPR polymorphisms on brain structure; existing data suggest that the relationship is complex and cannot be deduced from the independent effects of the two genes [26, 42, 43].

### Study aims and hypotheses

In this study, we assessed the effects of Val66Met and 5-HTTLPR polymorphisms (tri-allelic classification) on cortical thickness in cingulate, frontal and parahippocampal regions as well as the insula – areas most-consistently modulated by these polymorphysims. This is an extension of previous work in the same cohort where we found thicker frontal poles and thinner transverse temporal gyri in MDD compared with control groups [15]. We also assessed the influence of Val66Met and 5-HTTLPR polymorphisms on para/limbic structures (thalamus, caudate, putamen, pallidum, hippocampus and amygdala). To maximize power, and given the paucity of data on the effects of genetic polymorphisms on cortical thickness, all analyses were first carried out on groups (MDD and control) collapsed. Exploratory analyses (due to the limited sample sizes in specific allele groups) were then carried out on the MDD group; this was not possible in controls due to the small sample size.

Consistent with previous work, we expected smaller hippocampal volumes in Met allele (vs. Val/Val) carriers. We expected volumetric reductions in other para/limbic structures as well as cortical thickness decreases in Met carries, which would be more pronounced in the MDD cohort. Given the mixed data regarding the influence of the 5-HTTLPR polymorphism on brain architecture, directional hypotheses with respect to this polymorphism were difficult to make. However, it is possible that L_A_/L_A_ homozygotes would exhibit thickest cortices and greatest structural volumes, though this may not necessarily be true in the MDD cohort.

## Methods

### Participants

Consistent with previous work [15], 43 adults with a primary MDD diagnosis were recruited (Structured Clinical Interview for DSM IV-TR Diagnoses [SCID]-assessed). All patients had a Hamilton Rating Scale for Depression (HAMD_17_) score of ≥18 [44]. Notable exclusion criteria were: bipolar or anxiety disorder diagnosis (sub-threshold anxiety was not an exclusion criterion), psychosis history, current (<6 months) substance abuse/dependence, neurological or eating disorders, unstable medical condition, significant suicide risk and magnetic resonance imaging (MRI) contraindications. Participants were free of psychotropic drugs for at least three weeks at the time of neuroimaging. Fifteen healthy controls with no psychiatric history were also tested. Informed consent was obtained from all participants in accordance with the Conjoint Health Research Ethics Board at the University of Calgary.

### Magnetic resonance imaging (MRI)

Images were collected at the Seaman Family MR Centre (Foothills Hospital, University of Calgary) with a 3 T General Electric scanner (Signa LX, Waukesha, WI, USA) using an eight-channel head coil. A T1-weighted magnetization prepared rapid acquisition gradient echo (MPRAGE) image was acquired (TR = 8.3 ms; TE = 1.8 ms; flip angle = 20°; voxels = 0.5 × 0.5 × 1 mm; 1 mm slice thickness; 176 slices).

### Cortical thickness & automatic segmentation

Cortical thickness analyses were carried out using FreeSurfer software (http://surfer.nmr.mgh.harvard.edu), as previously described [15]. Briefly, T1 images were intensity-normalized, after which a skull-stripping procedure was applied. Images were segmented using an estimation of the structure of the grey-white interface. Each scan was covered with a triangular tessellation and inflated (for a smooth spherical representation of the grey-white interface and pial surface). Inflated scans were aligned to FreeSurfer’s default reference template via a 2D warp based on cortical folding patterns. Once smoothed (circularly symmetric Gaussian kernel), sulci and gyri curvature patterns were aligned and average cortical thickness was measured at each surface point. A uniform surface-based spherical coordinate system was created by transforming the reconstructed surfaces into parameterizable surfaces. An averaging procedure (50 iterations) was applied to smooth the surface and the reconstructed pial surface was refined with a deformable surface algorithm. Data was again aligned on a common spherical coordinate system. Cortical thickness was determined by measuring and averaging the distance between the grey/white matter boundary and pial surfaces. The following regions were assessed: a) cingulate regions (rostral anterior cingulate, caudal anterior cingulate, posterior cingulate and isthmus cingulate in both hemispheres), b) PFC/orbital regions (frontal pole, lateral and medial OBF as well as parsorbitalis cortices in both hemispheres), c) fronto-lateral regions (superior frontal, rostral middle frontal, parsopercularis, parstriangularis and caudal middle frontal cortices in both hemispheres), d) parahippocampal regions (entorhinal and parahippocampal cortices in both hemispheres) and e) the insula (in both hemispheres).

FreeSurfer was also used to obtain subcortical volume measures. A detailed description of the segmentation procedure can be found elsewhere [45]. Briefly, a training set is used to generate a probabilistic brain atlas that acts as a template used to assign labels to each voxel in an image. This probabilistic atlas estimates the probability with which particular structures are located throughout the brain, and acts as a Bayesian prior that can be used to assign structure labels to each voxel. Along with the probabilities given by the atlas, structures are also distinguished by assessing image intensity for specific tissue classes and the spatial location in relation to other structures. Volumetric assessments were carried out on the following: thalamus proper, caudate, putamen, pallidum, hippocampus and amygdala (both hemispheres).

### Genetic assessments

#### 5-HTTLPR

Either a 484 or 528 bp fragment was generated using a forward 5′-GGCGTTGCCGCTCTGAATGC and reverse 5′-GAGGGACTGAGCTGGACAACCAC primer [46]. Genes were amplified using AccuPrime GC-Rich DNA polymerase (Invitrogen, Carlsbad, CA, USA). The 25 μL amplification mixture contained 100 ng of genomic DNA, 0.2 μM of each primer, 1U of AccuPrime GC-Rich DNA polymerase and 1X AccuPrime GC-Rich Buffer A. The cycling conditions were: a) initial denaturation at 95 °C for 3 min followed by 7 cycles at 95 °C for 30 s, 68 °C for 30 s and 72 °C for 1 min; b) 7 cycles at 95 °C for 30 s, 67 °C for 30s and 72 °C for 1 min; c) 7 cycles at 95 °C for 30s, 66 °C for 30 s and 72 °C for 1 min. Final extension was at 72 °C for 10 min. The final uncut product was run on 1.5 % agarose gel at 50 V for 90 min; 12 μL of the PCR product was then cut using the restriction enzyme MspI (New England Biolabs Inc., Boston, MA, USA) for 3 h at 37 °C [47]. To separate bands, the cut product was run on a 4-20 % Tris/Borate/EDTA (TBE) gel at 14 mA for 100 min (Invitrogen, Carlsbad, CA, USA). The 5-HTTLPR is a 44 bp deletion in the promoter region at bp 1212–1255 (chromosome 17), resulting in a short (S) or long (L) allele. Within the extra 44 bp associated with the L allele, there is also a guanine to adenine polymorphism, resulting in L_G_ and L_A._ The band pattern for L_A_ was 340 bp, L_G_ was 166 + 174 bp and S was 297 bp [47].

#### Val66Met

A 113 bp fragment was amplified using a forward 5′-GAGGCTTGACATCATTGGCT and reverse primer 5′-CGTGTACAAGTCTGCGTCCT [48]. The 25 μL amplification mixture contained 50 ng of genomic DNA, 0.2 μM of each primer and 1X AccuStart Taq DNA polymerase (Quanta Biosciences, Gaithersburg, MD, USA). The cycling conditions were: a) initial denaturation at 95 °C for 2 min; b) 35 cycles at 94 °C for 30 s; c) 60 °C for 30 s; d) 72 °C for 30 s; e) final extension following the completion of the cycles at 72 °C for 5 min [49]. The 7.5 μL PCR product was cut with the restriction enzyme Eco 721 (Fermentas, Glen Burnie, MD, USA) for 3 h at 37 °C. The bands were separated using a 4-20 % TBE gel (Invitrogen, Carlsbad, CA, USA) at 14 mA for 60 min. The resulting banding pattern for the methionine and valine allele was 113 bp and two bands at 78 and 35 bp, respectively.

### Statistical analyses

MDD and Control groups were compared on clinical/demographic variables using one-way analyses of variance (ANOVAs) and Chi Square tests.

For the entire sample (MDD + Controls; *N* = 58), separate multivariate analyses of covariance (MANCOVAs; group as covariate) were carried out to assess the effects of genotype [Val66Met polymorphism: a) Val homozygotes (Val/Val) & b) Met allele carriers (Met/-); 5-HTTLPR polymorphysim: a) L_A_/L_A_ b) S/S (includes L_G_/S, L_G_/L_G_ & S/S carriers) & c) L_A_/S (includes L_A_/L_G_ & L_A_/S carriers) on cortical thickness in distinct regions. Specifically, MANCOVAs (separate MANCOVAs for Val66Met and 5-HTTLPR polymorphisms) were carried out on cortical thickness in 5 pre-specified regions (cingulate; PFC/orbital; fronto-lateral; parahippocampal; insula). An omnibus MANCOVA, where all 32 sub-regions were included as dependent variables, was also carried out. However, given that the separate MANCOVAs for each of the 5 regions were of primary interest (due to region-specific hypotheses), the omnibus MANCOVA was followed by the 5 separate MANCOVAs, regardless of statistical significance. Exploratory MANOVAs (as above, no group covariate) were carried out for the MDD group only (*N* = 43). Significant MANCOVAs/MANOVAs (*p* < .05, as well as trends: *p* < .1) were followed-up with univariate ANCOVAs/ANOVAs. Assessment of interactions between the Val66Met and 5-HTTLPR genotypes was not possible due to the small sample sizes for certain genotypes.

Finally, secondary one-way ANOVAs were also carried out to explore the effect of genotype on clinical variables only in the MDD group, namely HAMD_17_ scores, age of MDD onset or duration of the current depressive episode.

## Results

### Participant characteristics

There was a trend for an age difference (*p* = .051) but no differences in the distribution of sexes or genotypes between the MDD and control groups (Table 1). The addition of age as a covariate did not alter the morphometric findings (outlined below), as such, it was not included in the analyses presented (data not shown). The overall genotype frequencies of the Val66Met polymorphism (Val/Val = .78, Val/Met = .21, Met/Met = .017) were within the Hardy-Weinberg equilibrium (Chi^2^*p* = .85). The overall genotype frequencies of the 5-HTTLPR polymorphism (L_A_/L_A_ = .23, L_A_/L_G_ = .07, L_G_/S = .07, L_A_/S = .404, S/S = .23) were also within the Hardy-Weinberg equilibrium (Chi^2^*p* = .92). No differences existed on demographic variables between the genotypes (data not shown). Additionally, no differences were noted on clinical variables in the MDD group based on Val66Met or 5-HTTLPR polymorphisms.

**Table 1:** Characteristics of major depressive disorder (MDD) and healthy control (HC) groups

Characteristics	MDD	HC
N	43	15
Sex (F/M)	26/17	8/7
Age (yrs.)	30.3 ± 8.1	36.6 ± 11.1
5-HTTLPR polymorphysim^a^	L_A_/L_A_ homozygotes = 8; S/S homozygotes = 14; S/L_A_ heterozygotes = 20	L_A_/L_A_ homozygotes = 5; S/S homozygotes = 3; S/L_A_ heterozygotes = 7
Val66Met	Val/Val homozygotes = 35; Met/- carriers = 8 (Met/Met = 1; Val/Met = 7)	Val/Val homozygotes = 10; Met carriers = 5
Baseline HAMD_17_	21.4 ± 4.2	-
Duration of current MDE (yrs.)	4.4 ± 5.2	-
MDD onset (yrs.)	24.5 ± 10.9	-

*HAMD*_*17*_Hamilton depression rating scale, *MDD* major depressive disorder

Means ± SDs presented

^a^S/S homozygotes: S/S, L_G_/S & L_G_/L_G_; S/L_A_ heterozygotes: L_A_/S & L_A_/L_G_

### Effect of 5-HTTLPR alleles on cortical thickness & volumes of limbic structures

The omnibus MANCOVAs (all 32 sub-regions included as dependent variables) yielded no effect of 5-HTTLPR genotype on cortical thickness when MDD and control groups were collapsed [λ(62,44) = .76, *p =* .84]. Similarly, MANCOVAs were insignificant, when groups were collapsed, with respect to 5-HTTLPR genotype on cortical thickness within cingulate [λ(16,90) = .42, *p =* .98], parahippocampal [λ(8,98) = .87, *p =* .55], PFC/orbital [λ(16,90) = .87, *p =* .61], fronto-lateral regions [λ(20,86) = .97, *p =* .50] or the insula [λ(4,102) = 1.05, *p =* .34]. The omnibus MANOVA for 5-HTTLPR genotype on cortical thickness (i.e., all regions included) for the MDD group was significant [λ(62,18) = 2.85, *p =* .01], however, none of the follow-up univariate ANOVAs (for all 32 sub-regions) were found to be significant. Further, none of the 5 MANOVAs assessing cortical thickness per region were significant (cingulate [λ(16,62) = .45, *p =* .96], parahippocampal [λ(8,70) = 1.65, *p =* .13], PFC/orbital [λ(16,62) = 1.12, *p =* .36], fronto-lateral regions [λ(20,58) = .91, *p =* .58] or the insula [λ(4,74) = 1.03, *p =* .37]).

A weak trend for a significant MANCOVA was noted for 5-HTTLPR genotype on para/limbic structure volume when groups were collapsed [λ(24,86) = 1.44, *p =* .1]. Follow-up univariate ANOVAs yielded a main effect of 5-HTTLPR genotype on the right pallidum [F(2,54) = 3.43, *p* = .04], with a smaller volume in the L_A_/S heterozygotes versus the S/S homozygotes (*p* = .016; Additional file 1: Table S1A).

**Fig. 1:**
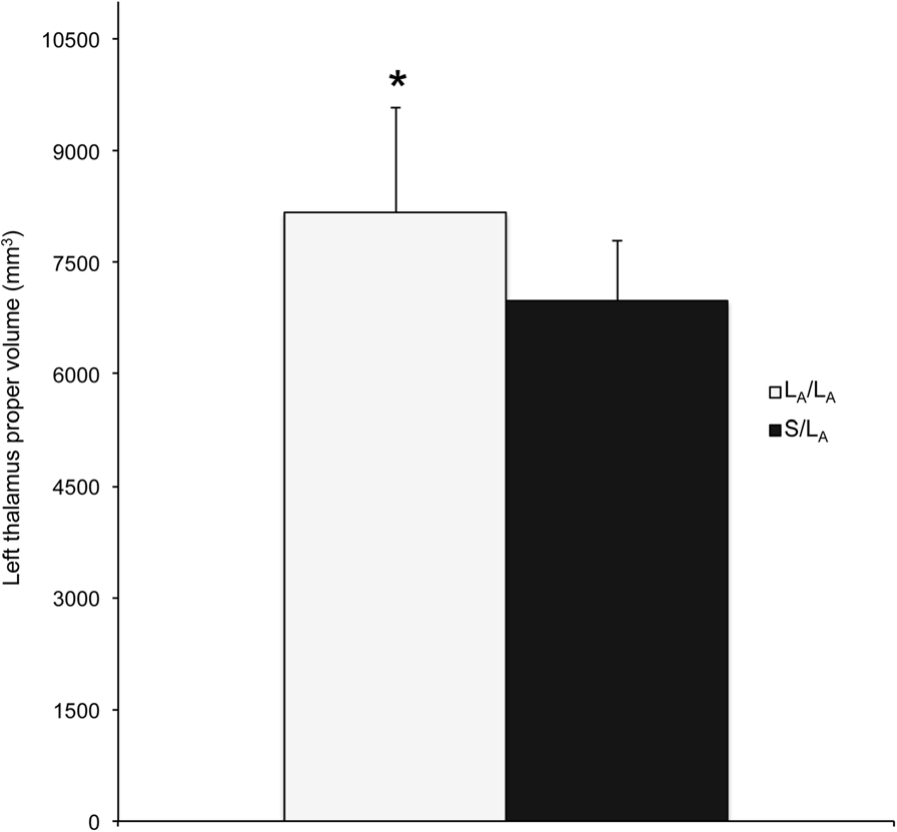
Greater (*p < .05) left thalamus proper volume in L_A_/L_A_ homozygotes versus S/L_A_ (L_A_/S or L_A_/L_G_) heterozygotes in the depressed cohort

**Fig. 2:**
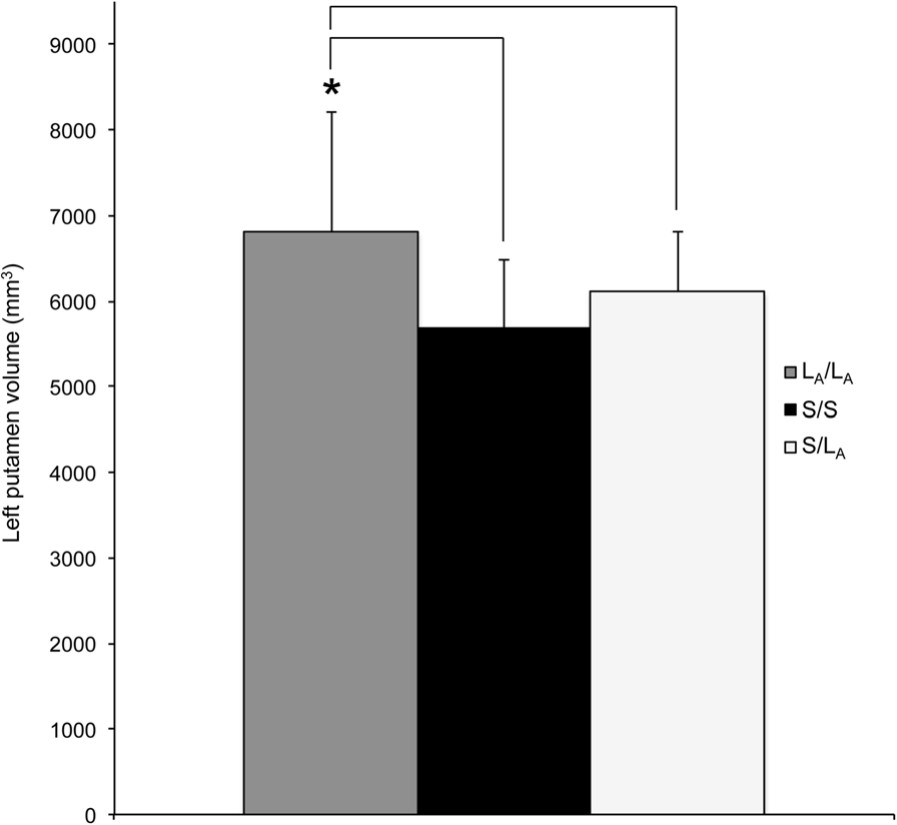
Greater (*p < .05) left putamen volumes in L_A_/L_A_ homozygotes versus both S/L_A_ (L_A_/S or L_A_/L_G_) heterozygotes and S/S (S/S, L_G_/S, L_G_/L_G_) homozygotes in the depressed cohort

A significant omnibus MANOVA was noted for 5-HTTLPR genotype on para/limbic structure volume for the MDD group [λ(24,56) = 1.93, *p =* .02]. Univariate ANOVAs yielded a main effect of 5-HTTLPR genotype on the left thalamus proper [F(2,41) = 6.47, *p* = .004] and left putamen [F(2,41) = 4.83, *p* = .013]. Follow-up comparisons indicated that the left thalamus proper was greater for L_A_/L_A_ homozygotes (*N* = 8; 8168.50, SD = 1407.38) compared with L_A_/S heterozygotes (*N* = 20; 6982.80, SD = 801.95; *p* = .005; Fig. 1). L_A_/L_A_ homozygotes (*N* = 8; 6805.38, SD = 934.92) also had a greater left putamen volume than S/S homozygotes (*N* = 14; 5691.57, SD = 879.75; *p* = .004) and L_A_/S heterozygotes (*N* = 20; 6107.90, SD = 700.34, *p* = .046; Fig. 2; Additional file 1: Table S1B).

### Effects of BDNF Val/66/Met polymorphisms on cortical thickness and volumes of limbic structures

The omnibus MANCOVA (all sub-regions included as dependent variables) yielded no effect of the Val66Met polymorphism (*N* = 13 Met/-; *N* = 45 Val/Val) on cortical thickness when groups were collapsed [λ(31,24) = .47, *p =* .98]. Similarly, the MANCOVAs were insignificant for the effects of the Val66Met polymorphism on cortical thickness in any of the five regions examined, when groups were collapsed [cingulate [λ(8,47) = .62, *p =* .76], parahippocampal [λ(4,51) = .70, *p =* .55], PFC/orbital [λ(8,47) = .52, *p =* .84], fronto-lateral regions [λ(10,45) = .69, *p =* .73], insula [λ(2,53) = 0.03, *p =* .88]. The omnibus MANOVA for Val66Met polymorphism effects on cortical thickness (all sub-regions) for the MDD group only was insignificant [λ(31,10) = .46, *p =* .95]; MANOVAs assessing cortical thickness per region were also insignificant (*p* values > .1).

The MANCOVA also yielded no Val66Met polymorphism effects on the volumes of para/limbic structures when groups were collapsed [λ(12,44) = .43, *p* = .94; Additional file 2: Table S2A). No effect of the Val66Met polymorphism on para/limbic structure volumes existed for the MDD group either [λ(12,30) = .35, *p* = .97]; exploratory assessments indicated no polymorphism differences in the hippocampus, where differences were hypothesized (*p* = .77, left hippocampus; *p* = .70, right hippocampus; Additional file 2: Table S2B).

## Discussion

This study examined the influence of 5-HTTLPR and Val66Met polymorphisms on cortical thickness in regions involved in emotion processing and cognitive control as well as para/limbic structure volumes in depressed individuals and healthy controls. No 5-HTTLPR genotype effect was found on cortical thickness in the examined regions when groups were collapsed or when only the MDD group was examined. A 5-HTTLPR genotype effect, however, existed on right pallidum volumes, with slightly smaller volumes in L_A_/S heterozygotes versus S/S homozygotes across the entire sample (a similar trend was noted compared with L_A_/L_A_ homozygotes: L_A_/S < L_A_/L_A_). More robust findings were greater left thalamus proper and putamen volumes in depressed L_A_/L_A_ homozygotes. No Val66Met polymorphism effects existed on cortical thickness in any of the examined regions or on para/limbic structure volumes.

### Cortical thickness and genotypes

Although a handful of studies have examined the effects of 5-HTTLPR polymorphisms on grey matter volume/density and structural volume, no studies, to our knowledge, have systematically assessed the effects of 5-HTTLPR polymorphisms on cortical thickness in MDD, and only a few have done so in non-depressed populations. We found no effect of 5-HTTLPR genotype on cortical thickness in our sample. This is consistent with the null results of another group that assessed the association between 5-HTTLPR genotype and cortical thickness in the ventrolateral and medial PFC in healthy females, although they did find an association between cortical thickness and biased attention to emotive cues, which interacted with genotype [50]. Another group, however, found a thinner ACC in healthy, non-depressed female S/S homozygotes compared with female L/L homozygotes and S/L heterozygotes in both females and males [51]. The latter study, however, had a larger sample (>100 participants), which allowed for the evaluation of gender influences.

We also found no Val66Met polymorphism effects on cortical thickness, consisted with results by another group in non-depressed adults [52]. However, others have noted greater grey matter density/volume across diffuse brain regions in Val/Val versus Met allele carriers [53], though predominantly in frontal regions ([30, 31, 54] but see [24]). Notably, the relation between the Val66Met polymorphism and cortical thickness may be influenced by factors such as past trauma [32, 55] and age [52].

Despite our negative findings, it is not possible to rule out the role of polymorphisms on cortical thickness in MDD (and general population). However, a larger cohort is required as these influences may be subtle. In our previous study using a comparable sample, childhood trauma was shown to effect cortical thickness [15], suggesting that early adversity may play a greater role on brain morphometry than Val66Met and 5-HTTLPR polymorphisms. As our sample was limited, we were underpowered to assess the interaction between genotype and past trauma on cortical thickness and volumetric measures in the current study; though this is certainly worthy of investigation. Additionally, a larger cohort would allow for the assessment of possible interactive effects between MDD features and subtypes, age and sex with genotype (epigenetic effect) as well as gene-by-gene interactions (epistasis) on cortical thickness.

### Subcortical structural volumes and genotypes

We found slightly smaller right pallidum volumes in L_A_/S (L_A_/S, L_A_/L_G_) heterozygotes versus S/S (S/S, S/L_G_, L_G_/L_G_) homozygotes when MDD and healthy control groups were collapsed. Previously, S/L heterozygotes have been shown to exhibit increased white matter lesions in geriatric depression and lower central 5-HTT availability [35, 56]. However, to our knowledge, no published data on 5-HTTLPR polymorphism effects on pallidum structure exist, thus, our findings require independent replication. The ventral pallidum is a convergent point of several brain areas implicated in reward and cognitive processing (e.g. OBF, PFC and infralimbic cortex, amygdala, hypothalamus, ventral tegmental area); its outputs re-enter corticolimbic loops via the medial PFC and thalamus (which then relay projections to the PFC). As such, the ventral pallidum is implicated in regulating motivation and reward – domains of frequent study in the context of MDD [57]. While untangling why altered 5-HTT expression may be associated with pallidal volume alterations is difficult, 5-HT transmission has been shown to play a neuromodulatory role in the ventral pallidum (5-HTT expression differences may thus alter pallidal function and, ultimately, its structure). Although the ventral pallidum largely consists of cholinergic and GABAergic neurons, dorsal raphe 5-HT projections modulate their transmission (likely via 5-HT_1B/1A_ receptors [58]). However, the involvement of the 5-HT system in the pallidum warrants further study.

It is somewhat surprising that we found no other 5-HTTLPR polymorphism effects on other para/limbic structures when groups were collapsed, as previous studies report volumetric changes in the amygdala and hippocampus in relation to the polymorphism. However, these data are mixed, with some groups noting greater amygdala volumes in S/S homozygotes or S allele carriers [41, 51], while others have found the opposite, with S allele carriers (vs. L/L homozygotes) exhibiting smaller volumes [37, 59, 60]. While methodological differences (e.g. segmentation methods; structural boundaries) may account for some of these differences, other factors likely play a role – participant ethnicity, mental health status and gender all appear to contribute to the relation between genetics and brain morphometry. Similar explanations may account for the variable reports with respect to the influence of the 5-HTTLPR polymorphysim on hippocampal volume [37–39]. While it is feasible that an 5-HTTLPR polymorphism effect on hippocampus and amygdala volume may have emerged with a larger sample, our null findings are consistent with others [61–63].

When the MDD group was assessed separately, a larger left thalamus proper were found in depressed L_A_/L_A_ homozygotes versus S/L heterozygotes. Our findings are inconsistent with those of Young et al., who reported an enlarged thalamus in S/S homozygotes (though, in the depressed cohort, thalamic volume increases existed independent of genotype) [64]. Midline thalamic regions have dense 5-HTT-containing fibers, as such, it is feasible that 5-HTTLPR modulations would affect the neural environment and, by extension, thalamic morphometry [65]. The thalamus plays an important role in regulating the expression and experience of emotion and has been implicated in MDD pathophysiology [64], however, the interactive effect between 5-HTTLPR polymorphism and MDD on the volume of thalamus, requires further study.

Similarly, a main effect of 5-HTTLPR genotype existed on the left putamen in the MDD cohort, with greatest volumes in depressed L_A_/L_A_ homozygotes. Though preclinical work suggests that the major input to the caudate-putamen is dopaminergic, it is also receives 5-HT projections [66]. Interestingly, greater 5-HTT densities in the putamen of healthy Caucasian L_A_/L_A_ homozygotes have been found [47]. This supports the idea that 5-HT function modulations by virtue of the genotype could influence both the activity and the structure of the putamen. Radua et al., found that right putamen grey matter volume was smaller in healthy individuals who were both COMT-Met and 5-HTTLPR S allele carriers, or both COMT-Val and 5-HTTLPR L/L homozygotes [61]. Another group found that in healthy individuals, the number of daylight minutes correlated negatively with 5-HHT expression in the putamen but only in S allele (not L/L) carriers. This suggests that S allele carriers may be more likely to exhibit dynamic changes in 5-HT activity/turnover, which may be associated with putamen volume changes [67]. Given the volumetric changes observed in depressed L_A_/L_A_ homozygotes, future studies may wish to examine the prognostic implications of this polymorphism.

Contrary to our hypotheses, we found no Val66Met polymorphism effects on hippocampal volume. Given that most evidence suggests decreased hippocampi in Met allele carriers (vs. Val/Val homozygotes) in both healthy controls and MDD patients [20–24], we expected comparable findings. With the exception of the Cardoner et al., (2013) study, however, all of the other studies consisted of 100+ participants, as such, it is possible that our comparatively small sample prevented the emergence of group differences. Additionally, we used an automatic segmentation approach, which may have also contributed to our null results [25]. Finally, a notable number of studies have found no hippocampal differences in relation to Val66Met polymorphisms [26, 27].

Despite the fact that this study is among the first to assess 5-HTTLPR and Val66Met polymorphisms effects on cortical thickness and para/limbic structure volume in depressed individuals, certain study limitations must be acknowledged. First, our sample was modest. This prevented us from assessing polymorphism interactions, the putative contribution of gender (and other factors, e.g. age of depression onset and childhood abuse history, and their possible moderating effects) on brain morphometry as well as examining polymorphism effects in the control group; such analyses are recommended in comparable future work. Furthermore, although we only followed-up MANCOVAs/MANOVAs that were significant (*p* < .05) or tended toward significance (*p* < .1), which was a means of minimizing the number of follow-up comparisons, we did not strictly adjust for multiple comparisons. As such, our results should be treated as preliminary and warrant replication. Additionally, given that our control sample was limited, we could not assess group-by-genetic polymorphism interactions on brain morphometry, which is recommended in similar studies.

## Conclusion

In this study, we noted slightly smaller right pallidum volumes in S/L_A_ heterozygotes versus S/S homozygotes when groups were collapsed, suggesting an influence of 5-HTTLPR on pallidum volume. The most pronounced 5-HTTLPR genotype effects emerged when the MDD group was assessed independently. Greater left thalamus and putamen volumes were found in L_A_/L_A_ homozygotes, consistent with hypotheses. These data point to the possibility that 5-HT activity alterations may be associated with morphometric changes in regions implicated in emotional and reward processing in MDD. No 5-HTTLPR genotype effects existed on cortical thickness; the same was true for the Val66Met polymorphism. Given that genetic imaging studies require large samples, multicentre involvement and data sharing will go a long way in advancing this field in the future.

### Ethics approval and consent to participate

The Conjoint Health Research Ethics Board (CHREB) at the University of Calgary approved this study (ethics ID: E-21396). Written informed consent was obtained from all participants. No individual data was used in this manuscript (only aggregate data are presented).

### Availability of data and materials

For access to the data presented in this manuscript, please contact the corresponding author.

## Additional files

Additional file 1: Table S1. A: Volume (mm^3^) of para-/limbic structures in L_A_/L_A_, S/S and L_A_/S allele carriers in individuals with MDD (major depressive disorder) and healthy controls (HC), combined. B: Volume (mm^3^) of para-/limbic structures in L_A_/L_A_, S/S and L_A_/S allele carriers in individuals with MDD (major depressive disorder). (DOCX 49 kb) Additional file 2: Table S2. A: Volume (mm^3^) of para-/limbic structures in Val/Val and Met/- allele carriers in individuals with MDD (major depressive disorder) and healthy controls (HC), combined. B: Volume (mm^3^) of para-/limbic structures in Val/Val and Met/- allele carriers in individuals with MDD (major depressive disorder). (DOCX 38 kb)

## Abbreviations

5-HTT: serotonin (5-HT) transporter
5-HTTLPR: 5-HT transporter gene linked polymorphism
ACC: anterior cingulate cortex
BDNF: brain-derived neurotrophic factor
COMT-Val: catechol-o-methyl transferase-valine
DLPFC: dorsolateral prefrontal cortex
DNA: deoxyribonucleic acid
HAMD_17_: Hamiton Depression Ratings Scale (17 items)
HC: healthy controls
MANCOVA/MANOVA: multivariate analysis of covariance/variance
MDD: major depressive disorder
MRI: magnetic resonance imaging
OBF: orbitofrontal
PCR: polymerase chain reaction
PFC: prefrontal cortex
TBE: tris-acetate-EDTA (ethylenediaminetetraacetic acid)
TE: echo time
TR: repetition time
Val66Met: valine to methionine substitution at codon 66
