# Supplementary material for: The influence of 5-HTTLPR and Val66Met polymorphisms on cortical thickness and volume in limbic and paralimbic regions in depression: a preliminary study
Source: BMC Psychiatry. 2016 Mar 15;16:61. doi: 10.1186/s12888-016-0777-x (PMC4791880; doi:10.1186/s12888-016-0777-x)
Supplement: Additional file 1: Table S1. — A: Volume (mm3) of para-/limbic structures in LA/LA, S/S and LA/S allele carriers in individuals with MDD (major depressive disorder) and healthy controls (HC), combined. B: Volume (mm3) of para-/limbic structures in LA/LA, S/S and LA/S allele carriers in individuals with MDD (major depressive disorder). (DOCX 49 kb) [file 12888_2016_777_MOESM1_ESM.docx]

**Additional file 1**

**Table S1A.** Volume (mm^3^) of para-/limbic structures in L_A_/L_A_, S/S and L_A_/S allele carriers in individuals with MDD (major depressive disorder) and healthy controls (HC), combined

| **Structure** | **L_A_/L_A_ (N=13)** | **S/S (N=17)** | **L_A_/S (N=27)** |
| --- | --- | --- | --- |
| Thalamus proper | 7623.5 ± 1441.8 (L)  7484.9 ± 1336.6 (R) | 7873.5 ± 1074.5 (L)  7206.4 ± 959.4 (R) | 7299.6 ± 1044.2 (L) 7016.9 ± 924.7 (R) |
| Caudate | 4030.8 ± 692.9 (L)  4067.9 ± 903.6 (R) | 3787.8 ± 592.3 (L)  3874.9 ± 627.6 (R) | 3694.2 ± 449.1 (L)  3669.7 ± 535.0 (R) |
| Putamen | 6665.0 ± 1022.1 (L)  6262.5 ± 896.5 (R) | 5934.6 ± 1119.2 (L)  5650.4 ± 1193.8 (R) | 6131.4 ± 657.0 (L)  5630.0 ± 743.5 (R) |
| Pallidum | 1671.9 ± 235.2 (L)  1526.3 ± 227.3 (R) | 1599.3 ± 323.1 (L)  1602.9 ± 269.4 (R) | 1482.5 ± 246.9 (L)  1409.4 ± 228.3 (R) |
| Hippocampus | 4504.1 ± 442.5 (L)  4673.2 ± 430.1 (R) | 4546.5 ± 576.2 (L)  4652.8 ± 522.2 (R) | 4476.6 ± 406.1 (L)  4480.2 ± 486.8 (R) |
| Amygdala | 1557.1 ± 262.5 (L)  1742.5 ± 242.0 (R) | 1454.1 ± 187.1 (L)  1641.5 ± 272.8 (R) | 1533.8 ± 269.5 (L)  1737.0 ± 271.3 (R) |

Means ± SDs presented

Note: S/S carries includes L_G_/S, L_G_/L_G_ & S/S carriers; L_A_/S carries includes L_A_/L_G_ & L_A_/S carriers

**Table S1B.** Volume (mm^3^) of para-/limbic structures in L_A_/L_A_, S/S and L_A_/S allele carriers in individuals with MDD (major depressive disorder)

| **Structure** | **L_A_/L_A_ (N=8)** | **S/S (N=14)** | **L_A_/S (N=20)** |
| --- | --- | --- | --- |
| Thalamus proper | 8168.5 ± 1407.4 (L)  7668.5 ± 1570.0 (R) | 7975.0 ± 882.9 (L)  7137.5 ± 770.7 (R) | 6982.8 ± 801.9 (L) 6790.1 ± 840.9 (R) |
| Caudate | 4111.3 ± 726.6 (L)  4115.0 ± 946.3 (R) | 3802.0 ± 539.2 (L)  3895.2 ± 547.4 (R) | 3635.9 ± 416.7 (L)  3594.9 ± 467.6 (R) |
| Putamen | 6805.4 ± 934.9 (L)  6212.8 ± 837.1 (R) | 5691.6 ± 879.8 (L)  5412.5 ± 966.2 (R) | 6107.9 ± 700.3 (L)  5691.8 ± 791.3 (R) |
| Pallidum | 1686.6 ± 235.6 (L)  1569.0 ± 228.1 (R) | 1537.6 ± 281.8 (L)  1582.9 ± 263.9 (R) | 1528.6 ± 258.0 (L)  1462.3 ± 229.0 (R) |
| Hippocampus | 4521.0 ± 252.0 (L)  4667.6 ± 387.8 (R) | 4484.4 ± 482.7 (L)  4569.6 ± 437.1 (R) | 4485.15 ± 440.4 (L)  4505.9 ± 551.8 (R) |
| Amygdala | 1546.1 ± 260.9 (L)  1794.5 ± 250.3 (R) | 1434.9 ± 177.1 (L)  1569.5 ± 217.4 (R) | 1554.4 ± 298.0 (L)  1734.2 ± 296.8 (R) |

Means ± SDs presented

Note: S/S carries includes L_G_/S, L_G_/L_G_ & S/S carriers; L_A_/S carries includes L_A_/L_G_ & L_A_/S carriers
