# Supplementary material for: The influence of 5-HTTLPR and Val66Met polymorphisms on cortical thickness and volume in limbic and paralimbic regions in depression: a preliminary study
Source: BMC Psychiatry. 2016 Mar 15;16:61. doi: 10.1186/s12888-016-0777-x (PMC4791880; doi:10.1186/s12888-016-0777-x)
Supplement: Additional file 2: Table S2. — A: Volume (mm3) of para-/limbic structures in Val/Val and Met/- allele carriers in individuals with MDD (major depressive disorder) and healthy controls (HC), combined. B: Volume (mm3) of para-/limbic structures in Val/Val and Met/- allele carriers in individuals with MDD (major depressive disorder). (DOCX 38 kb) [file 12888_2016_777_MOESM2_ESM.docx]

**Additional file 2**

**Table S2A.** Volume (mm^3^) of para-/limbic structures in Val/Val and Met/- allele carriers in individuals with MDD (major depressive disorder) and healthy controls (HC), combined

| **Structure** | **Val/Val (N=45)** | **Met/- (N=13)** |
| --- | --- | --- |
| Thalamus proper | 7603.8 ± 1077.9 (L)  7242.4 ± 1043.7 (R) | 7359.2 ± 1403.7 (L)  7025.3 ± 1022.7 (R) |
| Caudate | 3811.5 ± 510.0 (L)  3829.6 ± 631.4 (R) | 3741.0 ± 716.3 (L)  3784.7 ± 788.3 (R) |
| Putamen | 6228.5 ± 952.7 (L)  5771.0 ± 983.7 (R) | 6070.0 ± 803.7 (L)  5820.9 ± 824.1 (R) |
| Pallidum | 1559.0 ± 300.3 (L)  1499.1 ± 269.0 (R) | 1559.5 ± 160.5 (L)  1462.6 ± 176.6 (R) |
| Hippocampus | 4516.6 ± 458.6 (L)  4597.2 ± 505.5 (R) | 4465.3 ± 476.5 (L)  4520.2 ± 405.2 (R) |
| Amygdala | 1537.4 ± 266.2 (L)  1713.6 ± 278.7 (R) | 1460.6 ± 150.5 (L)  1715.6 ± 216.7 (R) |

Means ± SDs presented

**Table S2B.** Volume (mm^3^) of para-/limbic structures in Val/Val and Met/- allele carriers in individuals with MDD (major depressive disorder)

| **Structure** | **Val/Val (N=35)** | **Met/- (N=8)** |
| --- | --- | --- |
| Thalamus proper | 7493.1 ± 1028.5 (L)  7091.1 ± 1034.0 (R) | 7774.1 ± 1299.5 (L)  7107.4 ± 1024.5 (R) |
| Caudate | 3778.2 ± 511.5 (L)  3795.3 ± 614.7 (R) | 3776.8 ± 676.8 (L)  3776.1 ± 680.4 (R) |
| Putamen | 6095.1 ± 881.4 (L)  5631.3 ± 866.9 (R) | 6133.0 ± 877.6 (L)  6013.0 ± 905.2 (R) |
| Pallidum | 1552.8 ± 274.9 (L)  1511.8 ± 256.8 (R) | 1590.0 ± 195.6 (L)  1546.5 ± 166.4 (R) |
| Hippocampus | 4484.9 ± 418.1 (L)  4550.2 ± 494.3 (R) | 4532.9 ± 416.6 (L)  4624.5 ± 413.9 (R) |
| Amygdala | 1520.5 ± 278.8 (L)  1687.5 ± 276.7 (R) | 1515.3 ± 139.1 (L)  1738.1 ± 268.5 (R) |

Means ± SDs presented
